# Effectiveness of navigator-based prospective motion correction in MPRAGE data acquired at 3T

**DOI:** 10.1371/journal.pone.0199372

**Published:** 2018-06-28

**Authors:** Joelle E. Sarlls, Francois Lalonde, Dan Rettmann, Ajit Shankaranarayanan, Vinai Roopchansingh, S. Lalith Talagala

**Affiliations:** 1 NIH MRI Research Facility, National Institute of Neurological Disorders and Stroke, National Institutes of Health, Bethesda, Maryland, United States of America; 2 Functional MRI Facility, National Institute of Mental Health, National Institutes of Health, Bethesda, Maryland, United States of America; 3 GE Healthcare, Rochester, MN, United States of America; 4 GE Healthcare, Menlo Park, CA, United States of America; Public Library of Science, UNITED KINGDOM

## Abstract

In MRI, subject motion results in image artifacts. High-resolution 3D scans, like MPRAGE, are particularly susceptible to motion because of long scan times and acquisition of data over multiple-shots. Such motion related artifacts have been shown to cause a bias in cortical measures extracted from segmentation of high-resolution MPRAGE images. Prospective motion correction (PMC) techniques have been developed to help mitigate artifacts due to subject motion. In this work, high-resolution MPRAGE images are acquired during intentional head motion to evaluate the effectiveness of navigator-based PMC techniques to improve both the accuracy and reproducibility of cortical morphometry measures obtained from image segmentation. The contribution of reacquiring segments of k-space affected by motion to the overall performance of PMC is assessed. Additionally, the effect of subject motion on subcortical structure volumes is investigated. In the presence of head motion, navigator-based PMC is shown to improve both the accuracy and reproducibility of cortical and subcortical measures. It is shown that reacquiring segments of *k*-space data that are corrupted by motion is an essential part of navigator-based PMC performance. Subcortical structure volumes are not affected by motion in the same way as cortical measures; there is not a consistent underestimation.

## Introduction

Subject motion has long been an obstacle in MRI for producing high-resolution images that are free from artifacts, even more so for pediatric and patient populations. Because high-resolution MRI requires multi-shot acquisitions, subject motion between shots will cause inconsistencies in the *k*-space data, which results in image artifacts. Nonetheless, many researchers seek to perform studies utilizing high-resolution structural MRI to extract more accurate information about the thickness and volume of the cortex, as well as the volumes of subcortical structures. Typically, this is achieved by acquiring and segmenting high-resolution, ~1mm^3^, MPRAGE images [1]. In a recent study, Reuter et. al. showed that the effects of motion in such images will not just result in poor image quality from artifacts, but also bias the estimates of cortical volume and thickness [2].

Prospective motion correction (PMC) techniques are becoming increasingly available to improve the quality of high-resolution anatomical brain MRI. Multiple strategies have already been employed to mitigate effects of subject motion during MRI [3]. One approach is to use external hardware, like cameras, to monitor subject motion and feed the head position information back to the MRI scanner [4–6]. Others track head motion utilizing navigator data collected within the MR acquisition [7–11]. One such technique, PROMO [12], uses motion estimates from navigator data to adjust the imaging acquisition in every TR. PROMO is ideal for studies of pediatric or adult patient populations as no additional hardware needs to be placed on the subject [13]. When utilized within an MPRAGE sequence, PROMO has been previously shown to reduce errors in cortical surface reconstructions [12, 14], as well as remove bias in total gray matter volume and cortical thickness estimates for moving subjects [15, 16]. Navigator-based PMC techniques can employ two strategies to improve image quality: 1) updating the gradients and RF pulses to ensure excitation and acquisition of the same brain slice/slab and imaging field-of-view (FOV) in every TR (hereafter referred to as ‘FOV-update’), and 2) reacquisition of k-space data in which excessive motion has occurred between navigators acquired immediately before and after a particular k-space segment (hereafter referred to as ‘Reacquisition’). However, reacquisition of data adds extra scan time, increasing with increased subject movement, which is undesirable for pediatric or patient studies where scan time is limited. In this study, we investigate the relative contribution of the FOV-update and Reacquisition used in navigator-based PMC to improve accuracy in both cortical and subcortical morphometry measures in moving subjects. In addition, we investigate the improvement in reproducibility of those measures when using PMC.

## Methods

### Data acquisition

All data were collected on a 3T MR750 MRI system (GE Healthcare, Milwaukee, WI) with gradients capable of 50 mT/m amplitude and 200 T/m/s slew rate and a 32-channel receive-only array coil. Informed written consent was obtained from all subjects under an NINDS Institutional Review Board approved research protocol for this study. Data were acquired on 20 healthy young adult subjects using a 3D MPRAGE sequence with PMC and the following scan parameters: TE/TR = 3.4/8 ms, TI = 1150 ms, FA = 7°, FOV = 256 mm, resolution = 1x1x1 mm, ARC (parallel imaging factor) = 2, scan time = 6:48. In this work, PMC was accomplished using PROMO [12]. In PROMO, at the beginning of every TR, five sets of navigator images are acquired in the three orthogonal planes using 8° flip angle and single-shot spiral readout in approximately 14ms per set. The navigator images are registered to the navigators obtained at the start of the scan to estimate the head position using an extended Kalman filter algorithm. The new head position is then fed back to the sequence to update the FOV [12]. In addition, for any TR interval in which motion is detected to be greater than a preset threshold of ≥1 mm or degree, the corresponding *k*-space segment is tagged for reacquisition at the end of the scan.

In this work, two studies were conducted. In the 1^st^ study, the relative contribution of the FOV-update and Reacquisition used for navigator-based PMC to improve accuracy in both cortical and subcortical morphometry measures in moving subjects was evaluated. Data were acquired on 10 subjects who were instructed to perform a figure-eight motion with their nose when cued, for 10 seconds, 5 times during the scan. The motion cues were spaced evenly throughout the scan in a way to affect the center of k-space data. The cued movement ensured that the same segments of k-space data would be affected for all scans. In order to assess the efficacy of the two different parts of PMC jointly and independently, data were acquired under four conditions during intentional motion: 1) with PMC motion correction (i.e. FOV-update + Reacquisition); 2) FOV-update only; 3) Reacquisition only; 4) no PMC. Data were also acquired with PMC and no subject motion, which served as the control condition. A total of 5 MPRAGE data sets were acquired per subject.

In the 2^nd^ study, the reproducibility of both cortical and subcortical morphometry measures from moving subjects acquired with PMC, was evaluated. Data were acquired in 10 subjects instructed either not to move (control condition), or perform several figure-eight motions for 10 seconds with their nose every 60s, by their count. Separate intentional motion scans were acquired with and without PMC. Two scans were performed for each condition. A total of 6 MPRAGE data sets were acquired per subject.

### Analysis

All data sets were processed using FreeSurfer 5.3. Cortical and subcortical measures from both the surface-based and default streams were analyzed. From the surface-based stream, values of mean cortical thickness and total cortical volume of each brain lobe were calculated from the output parcellations, using the lobe mapping designations of ‘Desikan-Killiany’ ROIs [17]. From the default stream, subcortical structure volumes and the whole cortex volume were extracted. Briefly, both surface-based and default streams begin with registration to the MNI305 atlas[18] using an affine transformation. The two methods execute their own bias field correction scheme. Segmentations are based on prior probabilities generated from training sets composed of a series of manually segmented brains that were then registered to MNI305 surface space or volume space. The automated segmentation of surfaces and volumes has been shown to be highly correlated with manual segmentation [17, 19].

For the 1^st^ study, the accuracy of each morphometry measure estimated from each experimental condition was calculated as the normalized percent difference from the control condition, averaged across subjects. High accuracy is represented by smaller normalized percent difference. All measures were tested for significant differences using paired T-tests between the control and experimental conditions. Paired T-tests provide the maximum number of observations for each comparison given that not all data sets for all subjects and conditions will successfully complete the FreeSurfer segmentation algorithms. After Bonferroni correction for multiple comparisons, the level for significance was p < 0.00156 and p < 0.00625 for the surface-based and default stream morphometry measures, respectively.

For the 2^nd^ study, the reproducibility of each measurement was calculated as the average within-subject standard deviation (ws-sdev) normalized by the mean of the control condition measure:

normwsstdev=∑di22NM

where d*i* is the difference between the replicate scans, N is the number of subjects, and M is the mean of the control measure. High reproducibility is represented by smaller norm-ws-stdev.

## Results

**Fig 1:**
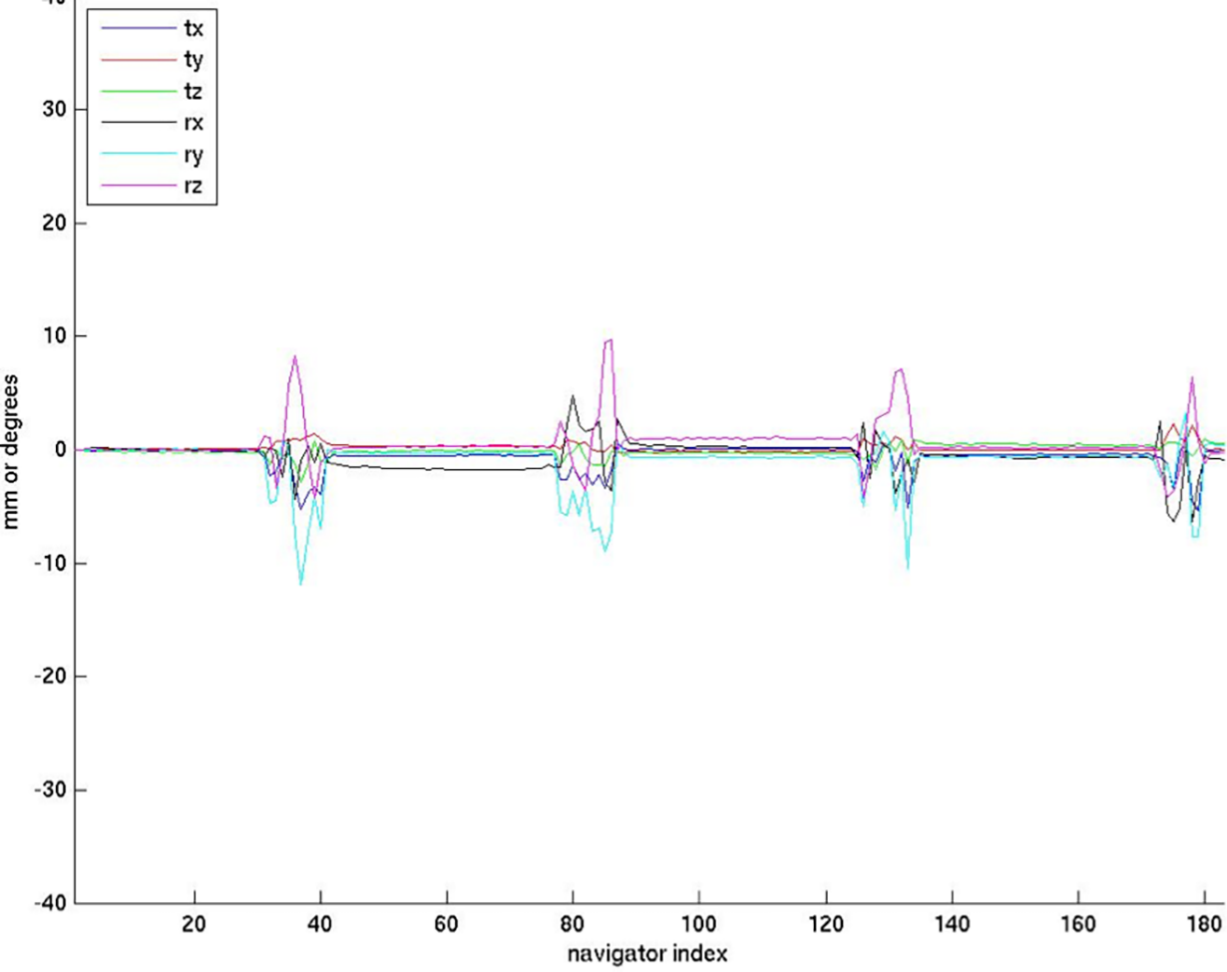
Plot of the estimated motion of a subject performing the figure-8 movement. Translations and rotations are plotted together, with the vertical axis units being mm or degrees.

**Fig 2:**
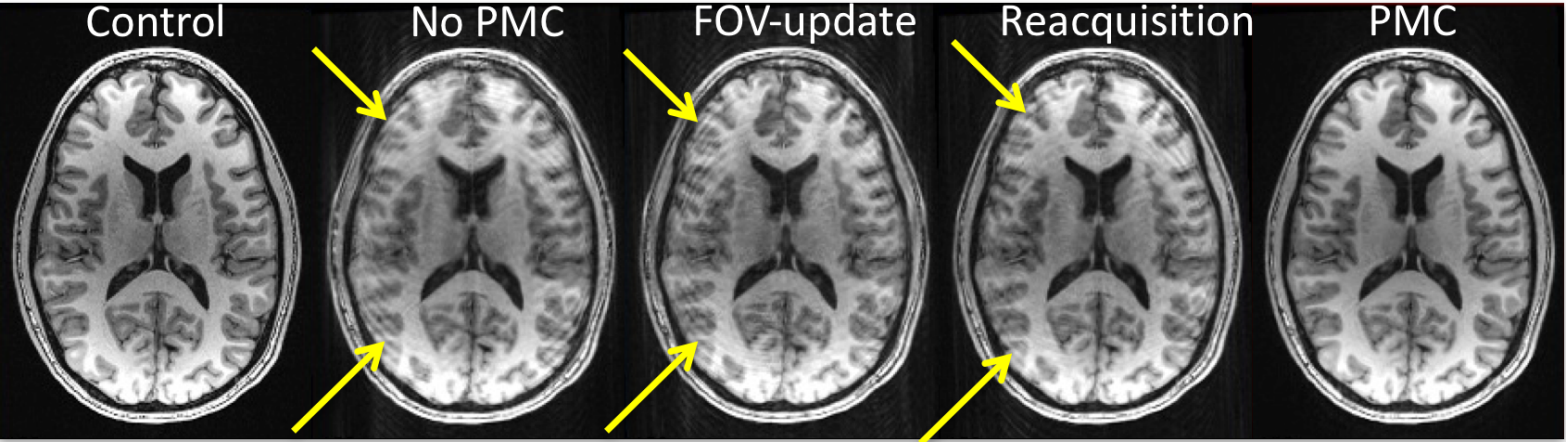
MPRAGE data acquired without motion and with motion under different experimental conditions. The yellow arrows indicate motion artifacts.

In the 1^st^ study, the within-subject motion was consistent between the experimental conditions. The range of translation and rotation from all subjects was ~1–10 mm and ~1–22°, respectively (see Fig 1). The additional time needed for reacquisition ranged from 0:51 to 1:57 (min:sec). There was no reacquistion triggered in the control condition of any subject, indicating there was no subject motion greater than 1 mm or degree. In the representative raw images shown in Fig 2, one can observe the level of artifact in the images acquired under various conditions. As expected, there are clear artifacts with subject motion and No PMC. The artifacts due to subject motion are similar in the FOV-update and Reacquistion conditions. Conversly, under the PMC condition, when both FOV-update and Reacquisition strategies are employed, there are no appreciable artifacts and the image quality is similar to the control condition.

**Fig 3:**
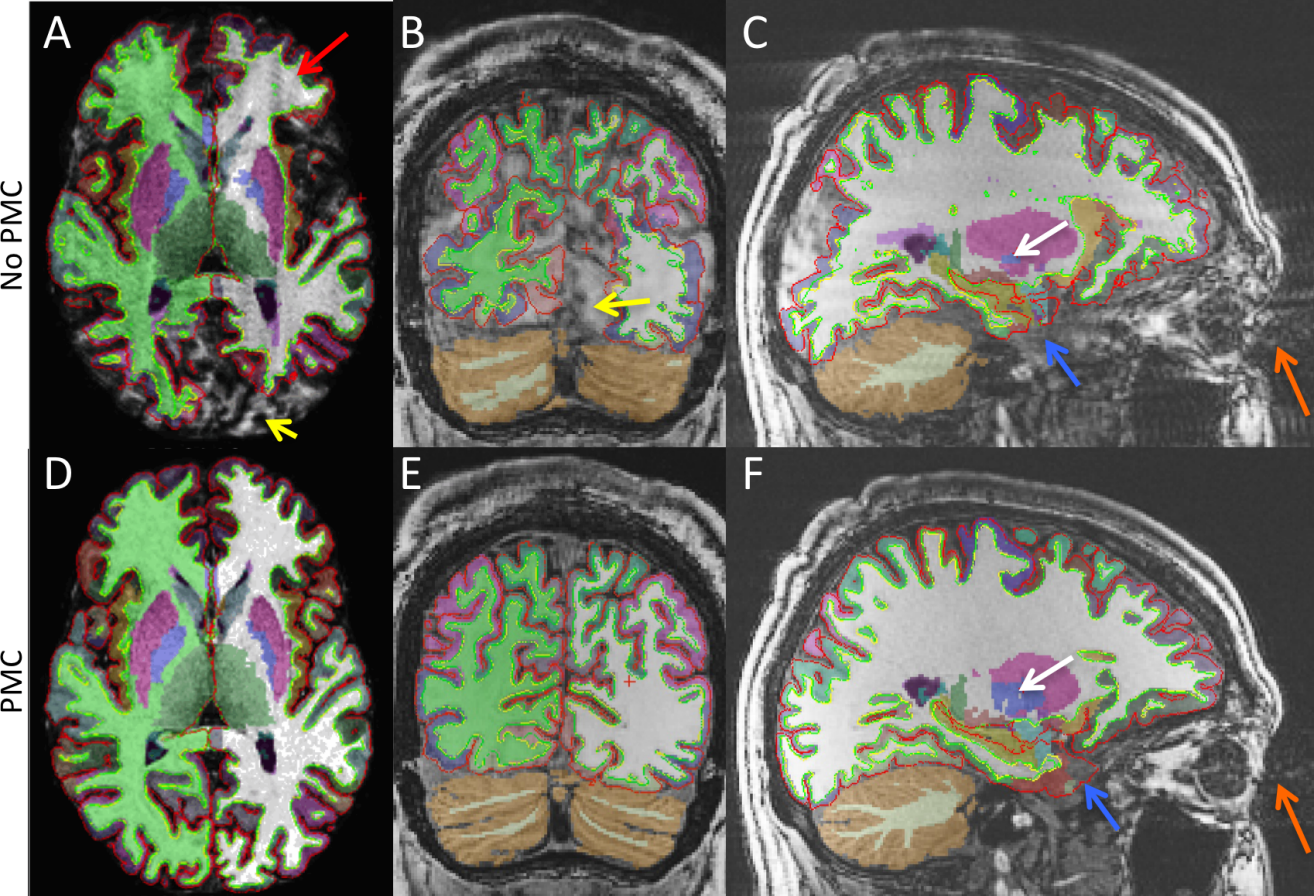
MPRAGE axial (A and D), coronal, (B and E), and sagittal (C and F), images overlaid with segmentation results. Top and bottom rows are the No PMC and PMC conditions, respectively. The red arrow indicates ringing artifacts due to motion. The yellow arrows indicate areas of poor segmentation due to motion artifacts. The orange arrows indicate artifacts in the phase-encode direction due to eye movement. The blue arrows indicate areas of poor segmentation in the temporal lobe due to eye movement artifacts. The white arrows indicate both the under and over estimation that occurs in subcortical structures due to motion artifacts.

Fig 3 shows example segmention results from FreeSurfer overlaid on anatomical images. As in Fig 2, images acquired with No PMC have ringing artifacts (red arrows) and segmentation errors (yellow arrows). These effects are not observed when PMC is applied during intentional subject motion. In addition, segmentation errors due to motion related eye movement (orange arrows) can be seen in the anterior temporal lobe in both the No PMC and PMC images (blue arrows). Fig 3C provides an example of both the under and over estimation that occurs in subcortical structures. Compared to the segmentation from PMC data, shown in Fig 3F, the Globus pallidus is smaller while the Putamen is larger (white arrows).

Due to the severity of motion related artifacts, FreeSurfer segmentation did not complete for all conditions in all subjects. FreeSurfer segmentation completed in at least 4 of the 5 data sets for 9 subjects. However, 2 data sets had segementation results that encompassed less than 50% of the brain and were not included in analysis. FreeSurfer segmentation did not complete for 3 of the 5 data sets for the 10^th^ subject; therefore, this subjects data was not included in the analysis. Thus, the total number of subjects analyzed for each condition were as follows: control = 9, no PMC = 7, FOV-update = 7, Reacquistion = 8, PMC = 9.

**Fig 4:**
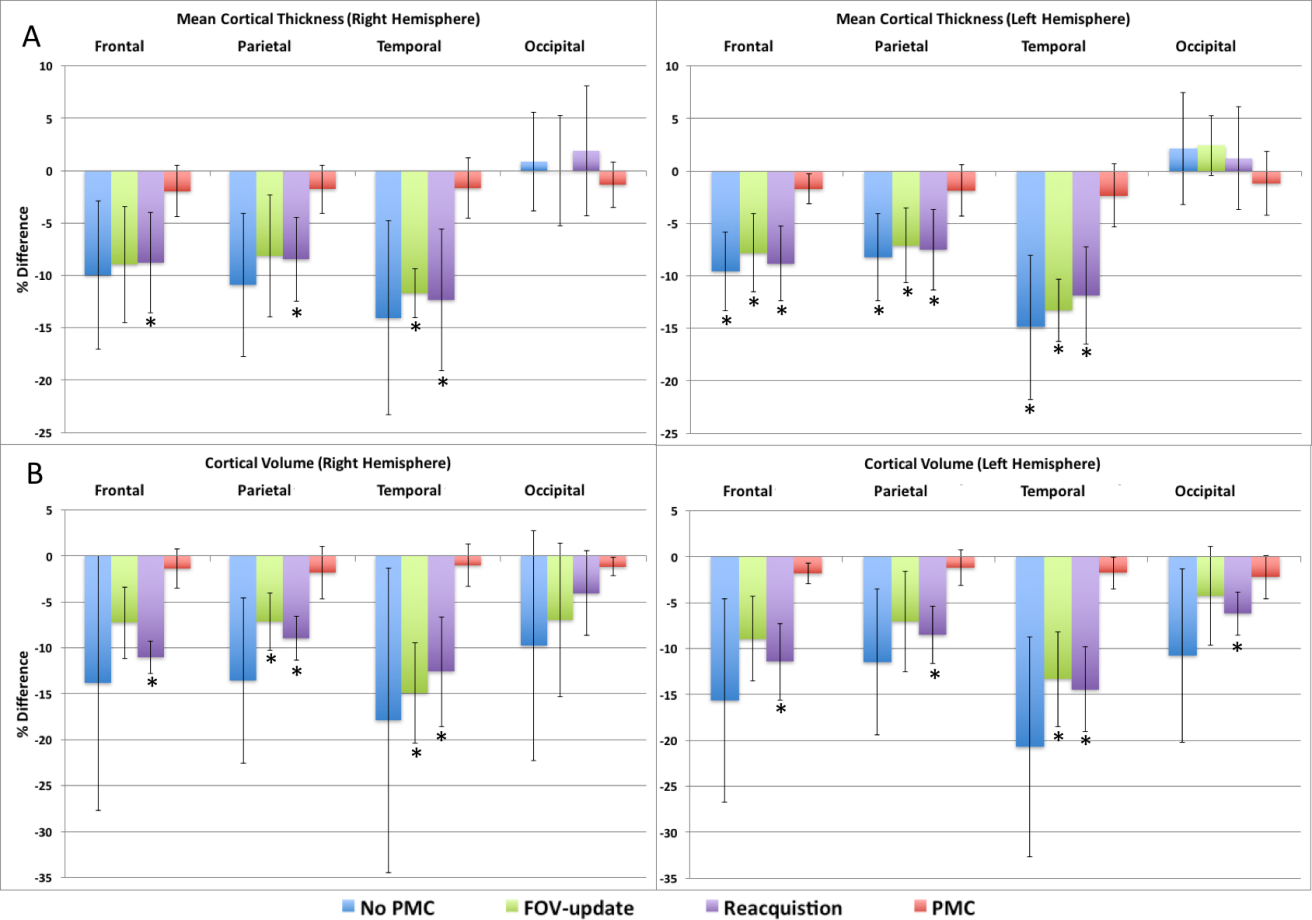
Plots of the average % difference of cortical measures extracted from the surface-based stream of FreeSurfer compared to the no movement, control, condition for mean cortical thickness (A), and total cortical volume (B). The * indicates significant difference compared to control condition at level p<0.00156.

Fig 4A and 4B show the accuracy of the mean cortical thickness and total cortical volume, respectively, for each lobe in the right and left hemispheres, as extracted from the surface-based stream of FreeSurfer. The mean thickness is greatly affected by subject motion with No PMC resulting in 8–15% bias of reduced thickness in the frontal, parietal, and temporal lobes compared to the no motion control. The accuracy of the mean thickness estimates for these brain lobes with either FOV-update or Reacquisition were similar to No PMC. The difference in mean cortical thickness compared to the control condition was statistically significant in the frontal, parietal, and temporal lobes of the left hemisphere for No PMC, FOV-update, and Reacquistion. The difference also reached significance in the same lobes of the right hemisphere for Reacquisition. The accuracy of mean cortical thickness in the occipital lobe is more variable, and does not result in a consistent bias for any condition. The utilization of PMC produced the most accurate results in all brain lobes, and the estimated mean cortical thickness was not significantly different than the control condition. Fig 4B shows that the total cortical volume is also greatly affect by subject motion with No PMC resulting in 9–21% bias of reduced volume. FOV-Update or Reacquisition produces a similar bias in total cortical volume as that of No PMC, and reaches statistically significant difference than the control in some brain lobes. Utilizing PMC produces the most accurate measures of total cortical volume, which are not significantly different from the control condition. These results indicate that reacquiring segments of k-space that have been tagged due to excessive motion is essential to the performance of navigator-based PMC. The bias in both mean cortical thickness and volume with subject motion and No PMC were similar to results reported by Reuter et. al [2].

**Fig 5:**
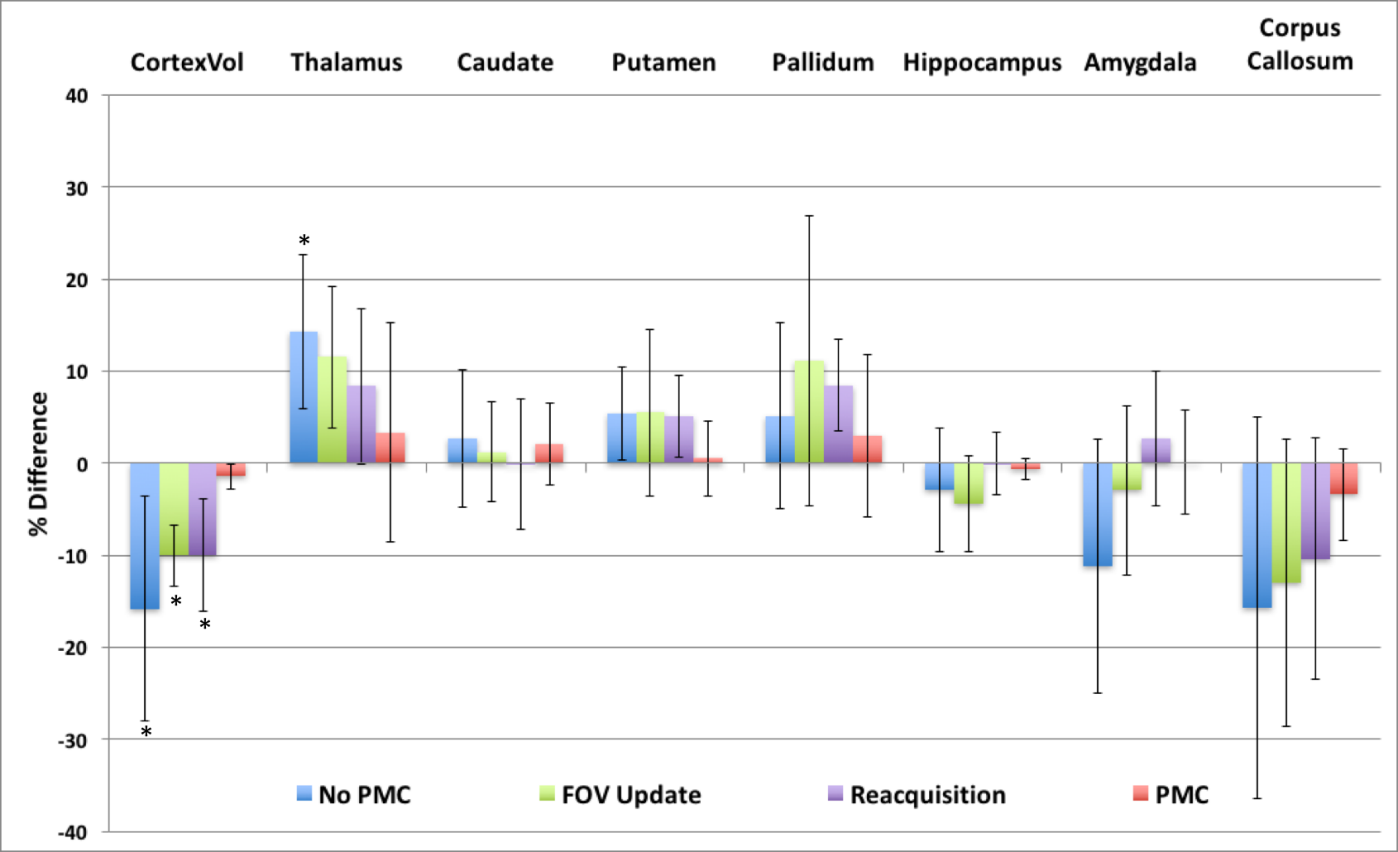
Plot of the average % difference of whole cortex and sub-cortical structure volumes extracted from the volume-based stream of FreeSurfer compared to the no movement, control, condition. The * indicates significant difference compared to control condition at level p<0.00625.

Fig 5 shows the accuracy of the whole cortex and subcortical structure volumes, as extracted from the default stream of FreeSurfer. The estimated cortex volume is significatnly affected when subjects are moving, indicated by the 15% mean decrease seen with No PMC. The accuracy of the cortex volume estimate is seen to improve with either FOV-update or Reacquisition alone, although still significantly different than the control condition. However, the average difference in the cortex volume compared to the control condition was lowest when PMC was utilized and not statistically significant from the control. Subcortical structure volumes are also affected by subject motion, but not in a consistent manner. The thalamus volume tends to be overestimated with motion, reaching statistical significance with No PMC. Conversely, the copus callosum volume tends to be underestimated, but is not statistically significant. In general, there is a large variation in the estimated volumes of subcortical structures across subjects, and thus the percent difference is not statistically significant in the majority of measures. These results indicate that there is not a consistent bias in subcortical structure volumes in data that is contaminated by motion artifacts like there is in cortical morphometry measures. However, Fig 5 does show that PMC has the smallest percent difference in volume estimates for all the subcortical structures compared to FOV-update or Reacquistion.

In the 2^nd^ study in which the subjects executed self-paced motion, the range of translation and rotation from all subjects was ~1–10 mm and ~1–20°, respectively. The additional scan time needed for PMC reacquisition ranged from 0:35 to 2:17 (min:sec). There was no reacquistion triggered in the control condition of any subject, indicating there was no subject motion of greater than 1 mm or degree. FreeSurfer segmentation was successful for all conditions in 9 of 10 subjects, thus 9 subjects were used for analysis.

**Fig 6:**
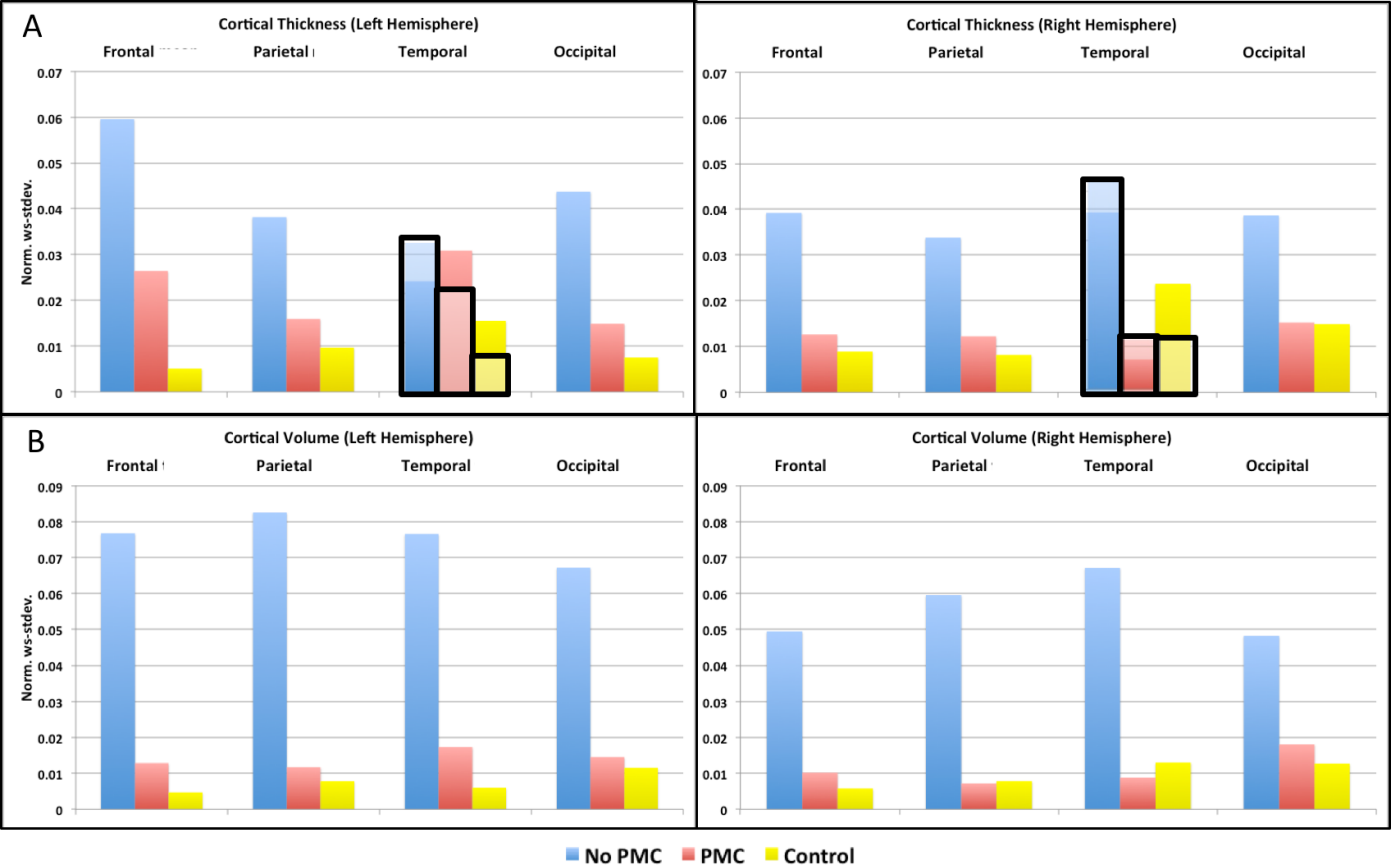
Graphs of the norm. ws-stdev. of the mean cortical thickness (A) and total cortical volume (B). In A, results excluding the temporal pole and entorhinal regions in the temporal lobes are highlighted and outlined in black.

Fig 6A and 6B show the reproducibility of the mean cortical thickness and total cortical volume, respectively, as extracted from the surface-based stream of FreeSurfer. In the presence of motion, PMC improves the reproducibility of both mean cortical thickness and total coritcal volume in both the left and right hemisphere of all brain lobes, except left temporal lobe. The anterior temporal lobe was the region affected by eye movement. In order to remove the image region exhibiting eye movement artifacts, the norm-ws-stdev. of the temporal lobe was also re-evaluated excluding the temporal pole and entorhinal cortex. These results are shown in Fig 6A and 6B as bars in the graph highlighted and outlined in black. When these anterior temporal lobe regions were excluded from the analysis, the PMC condition exhibits improved reproducibility compared to No PMC, similar to the other lobes. The reproducibility of morphometry measures from moving subjects acquired with PMC is similar to that of the control condition across the brain.

**Fig 7:**
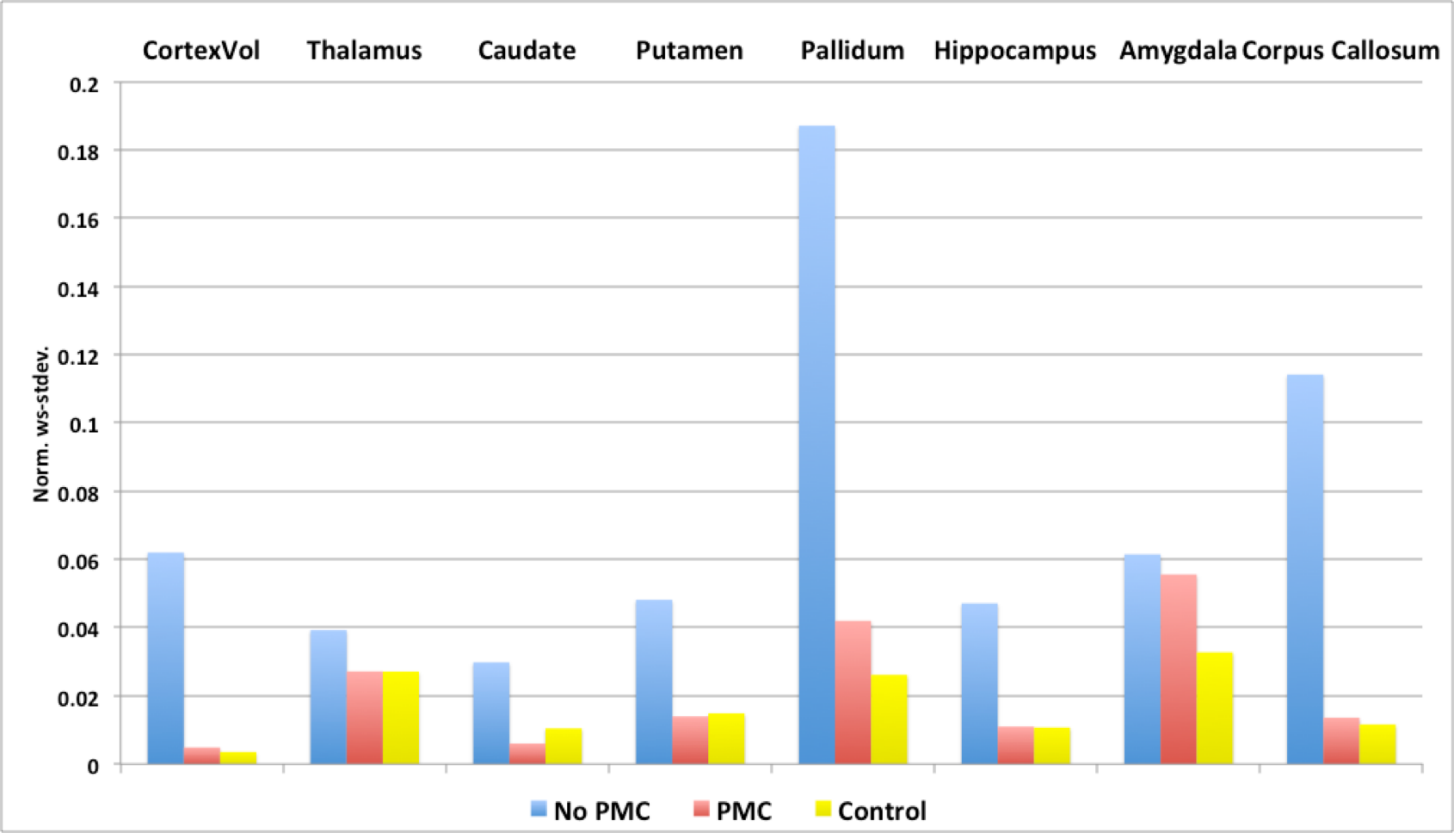
Graph of the norm. ws-stdev. of whole cortex and sub-cortical structure volumes.

Fig 7 shows the reproducibility of the whole cortex and subcortical structures volumes, as extracted from the default stream of FreeSurfer under different conditions. Subject motion causes the norm. ws-stdev. to be high, in otherwords reduces the reproducibility, in all subcortical structures and whole cortex volume. Utilizing PMC improves the reproducibility of the volume measure of all the subcortical structures, making them closer to the reproducibity in control images without motion.

## Discussion

The cortical measures obtained from both the surface-based and default streams of FreeSurfer segmentation of MPRAGE images were more accurate on the regional scale, as well as on the global scale of whole brain when utilizing PMC with subject motion (Figs 4 & 5). Subject motion produced a consistent bias of reduced cortical thickness and volume, as extracted from both the surface-based and default streams of FreeSurfer. These results are consistent with those of previously published works [11, 16]. Although there was some improvement in accuracy of cortical measures when either the FOV-update or Reacquisition were used, utilizing both PMC components provided even greater accuracy, resulting in no significant differences compared to a non-moving subject. These results indicate that reacquiring segments of k-space that have been tagged due to excessive motion is essential to the performance of navigator-based PMC.

Conversely, the subcortical structure volumes were affected by subject motion, but not with a cconsistent bias. The thalamus volume tended to be overestimated, whereas the corpus callosum tended to be underestimated. The other subcortical structure volumes were either increased or decreased depending on the subject, resulting in large standard deviations (Fig 5). Several plausible reasons exist for this discrepency between coritcal and subcortical measures. First, artifacts from subject head motion in 3D MPRAGE often present as ringing in the phase-encode direction and are most pronounced near edges. Therefore, the artifacts are stronger in the cortex than subcortical regions. Second, the cortex is only a few voxels wide in typical 1mm MPRAGE images, much thinner than subcortical structures. Thus, more easily affected by ringing artifacts. Lastly, the volume of subcortical structures during segmentation are dependant on each other. If one structures volume grows, another must shrink, as in the Globus pallidus and Putamen of Fig 3.

The reproducibility of cortical and subcortical structure volumes was improved when PMC was applied during intentional motion (Figs 6 & 7). The reproducibility in estimated cortical thickness also improved in frontal, parietal, and occipital lobes. However, the reproducibility of the cortical thickness measure in the temporal lobe of the left hemisphere was not improved. Upon further investigation, it was found that there was substantial variability in anterior temporal regions, most notably the temporal pole and entorhinal cortex. MPRAGE images under all scanning conditions often contain artifacts due to eye movement, which lead to poor segmentation in the affected regions (see Fig 3 blue arrows). Extracted measures from these regions may thus be less reliable. Removing the noted anterior temporal regions from the mean cortical thickness yields improved reproducibility when PMC was applied, as seen in the overlay of Fig 6A. The unreliable anterior temporal regions had little effect on the reproducibility of total cortical volume because their contribution to the total is small. To reduce the effects of eye movement on segmentation results, one can apply a saturation band over the eyes when acquiring MPRAGE data.

FreeSurfer segmentation completed for all subjects data sets under the control condition of no motion, as well as with PMC and subject motion. FreeSurfer segmentation did not complete for all subjects under the other conditions. Therefore, when assessing the accuracy of cortical measures the total number of subjects analyzed for each condition were unequal, varying from 7 to 9 subjects. A paired T-test was used to determine if each condition was statistically significantly different than the control. Although the difference in the number of subjects varies the statistical power between conditions, the test takes into account the number of subjects and thus the determined significance is valid for each condition. However, when assessing the reproducibility of cortical measures as the normalized within-subject standard deviation, we were able to analyze the same number of subjects for each condition.

There are potential limitations to this study. Subjects were asked to perform a figure eight motion because we believed it to be reproducible within subjects and such motion incorporates both translation and rotation. However, the figure eight motion is not representative of all subject head motions that occur when acquiring MRI data. In addition, subject movement was discrete. Results could differ for more continuous head movement. Further, because the data for the various motion correction conditions had to be acquired in separate scans, the movement for each condition was similar, but not identical. This may have introduced additional variability to the data.

This study utilized a 32-channel receive coil and the parallel imaging technique ARC [20]. Multi-channel receive coils may exacerbate artifacts due to subject motion. Utilizing a more robust reconstruction method that takes into account coil sensitivity may produce different results than presented here [21].

Our results indicate that the reacquisition part of navigator-based PMC is necessary to obtain segmentation measures similar to the control condition. In the current studies, the reacquisition phase typically required 15–30% more scan time. This could impact studies of pediatric or patient populations where longer time in the scanner is not tolerated well. In future work, one could evaluate if a smaller percentage of motion-corrupted data could be reacquired to provide sufficient improvement.

In this work, PROMO was used as the PMC technique. However, the results presented in this work would be equally applicable for other navigator-based PMC techniques as the rate of motion estimation and FOV updating would be similar. Prospective motion correction techniques that use external hardware have a much higher rate of motion estimation and FOV updating, therefore some results presented in this work may not be directly applicable. However, because reacquisition of motion corrupted data had a significant impact on the accuracy and reproducibility of measures extracted from segmentation in navigator-based PMC, it is a potentially useful area of exploration, as external hardware PMC techniques do not typically reacquire motion corrupted data.

## Conclusion

Both the accuracy and reproducibility of segmentation measures from high-resolution anatomical data are improved when prospective motion correction is utilized with moving subjects, without sacrificing subject comfort, and with a minimal increase in scan time. The accuracy of cortical thickness and volume was not significantly different from the control condition, of no motion, when utilizing prospective motion correction with a moving subject. Reacquiring k-space segments affected by motion was essential to PMC performance. In addition, it is shown that unlike cortical measures, subcortical structure volumes do not have a consistent bias due to subject motion.
